# The Prognostic Significance of CD47, CD68, and CD163 Expression Levels and Their Relationship with MLR and MAR in Locally Advanced and Oligometastatic Nasopharyngeal Carcinoma

**DOI:** 10.3390/diagnostics14232648

**Published:** 2024-11-24

**Authors:** Asim Armagan Aydin, Ramazan Oguz Yuceer, Senay Yildirim, Ahmet Unlu, Erkan Kayikcioglu, Murat Kocer

**Affiliations:** 1 Department of Clinical Oncology, Health Sciences University Antalya Education and Research Hospital, Antalya 07100, Turkey; md.ahmetunlu@gmail.com (A.U.); muratkocer71@hotmail.com (M.K.); 2 Department of Pathology, Cumhuriyet University School of Medicine, Sivas 58140, Turkey; r.yuceer66@hotmail.com; 3 Department of Pathology, Health Sciences University Antalya Education and Research Hospital, Antalya 07100, Turkey; dr_senayyildirim@hotmail.com; 4 Department of Clinical Oncology, Istinye University School of Medicine Liv Hospital, Istanbul 34517, Turkey; drkayikcioglu@yahoo.com

**Keywords:** nasopharyngeal carcinoma, CD47, CD68, CD163, tumor-associated macrophages, monocyte-to-lymphocyte ratio, monocyte-to-albumin ratio, prognosis, survival, immunotherapy

## Abstract

Background: This study aimed to assess the prognostic and predictive implications of CD47, CD68, and CD163, biomarkers of tumor-associated macrophages (TAMs), on the treatment efficacy and clinical outcomes of nasopharyngeal carcinoma (NPC). Additionally, the prognostic value of TAM-related indices, such as the monocyte-to-lymphocyte ratio (MLR) and monocyte-to-albumin ratio (MAR), was evaluated. Methods: A retrospective cohort of 54 patients with locally advanced or oligometastatic NPC treated with concurrent chemoradiotherapy (CCRT), with or without induction chemotherapy, was analyzed. Patients were categorized based on the cumulative expression scores for CD47, CD68, and CD163: negative/low (0–3 points) and high (4–6 points). MLR and MAR were also stratified as low MLR (<0.545) vs. high MLR (≥0.545) and low MAR (<16.145) vs. high MAR (≥16.145). The primary endpoint was overall survival (OS). Results: High CD47, CD68, and CD163 expression levels were correlated with advanced clinical stage, reduced CCRT response, and elevated MLR and MAR. These TAM biomarkers were linearly correlated with each other and with established risk factors such as advanced age and elevated EBV-DNA levels. Kaplan–Meier analysis revealed that patients with low TAM expression had significantly longer OS and progression-free survival (PFS) than those with high TAM expression. Multivariate analysis identified high CD163, MLR, and MAR levels as independent adverse prognostic factors for OS. Elevated MLR is an independent risk factor for both OS and PFS in patients with NPC. Conclusions: CD47, CD68, and CD163 are significant prognostic markers in NPC, with higher levels being associated with poorer OS and PFS. Elevated MLR and MAR values also predict worse outcomes, underscoring their value as prognostic tools. CD163 and MLR are particularly strong predictors, highlighting the crucial role of TAMs in NPC management and suggesting that CD163 is a potential therapeutic target within the immune checkpoint pathway.

## 1. Introduction

Nasopharyngeal carcinoma (NPC) is a distinct subtype of head and neck cancer with a decreasing incidence rate of approximately 1 per 100,000 annually in recent years [1]. Nevertheless, it is significantly more prevalent, six to nine times greater in Southeast Asia and North Africa than in other global regions [2,3,4]. NPC exhibits a male predilection, occurring three times more frequently in males than in females, and it is notably linked to latent Epstein–Barr virus (EBV) infection in endemic areas [3,4,5]. Furthermore, due to its distinct structural characteristics, including pronounced intratumoral heterogeneity, and its evaluation within the context of ecological and evolutionary processes, it represents a unique subgroup within head and neck tumors [6]. Despite advancements in radiotherapy techniques that reduce regional recurrence rates, NPC still exhibits a high propensity for distant metastases [7,8]. In early-stage NPC (T1-2, N0), definitive radiotherapy alone is an effective treatment [9,10]. However, for locally advanced and advanced stages (stage II to IVb), the current standard approach involves induction chemotherapy (gemcitabine–cisplatin) followed by concurrent chemoradiotherapy (CCRT) [9,11,12]. To mitigate the risk of systemic recurrence, ongoing efforts are exploring maintenance therapies such as capecitabine, following these established protocols [13]. Additionally, the emergence of immune checkpoint inhibitors, a prominent focus of cancer research in recent years, has been investigated in NPC. In a pivotal study by Mai et al., the PD-1 inhibitor toripalimab exhibited clinically significant survival advantages when integrated with standard chemotherapy for the treatment of recurrent or metastatic NPC, leading to FDA approval in late 2023 [14]. Understanding the interactions between NPC and the immune system, particularly the mechanisms of immune evasion and the potential of immune checkpoint inhibitors, is crucial for the development of effective therapies [15,16]. Ongoing research continues to explore new ways to harness the immune system to improve the outcomes of patients with NPC.

CD47, alternatively referred to as integrin-associated protein (IAP), is a transmembrane protein associated with binding proteins that plays a pivotal role in numerous physiological processes, including immune regulation, cell signaling, and the maintenance of tissue homeostasis [17,18]. CD47, known for its role as a “don’t eat me” signal on the cell surface, inhibits phagocytosis when it binds to signal regulatory protein alpha (SIRPα), which is expressed on macrophages [19,20]. This interaction prevents clearance of healthy cells through the immune system. This mechanism ensures the maintenance of immune tolerance and mitigates autoimmune reactions [21,22]. Increased expression of CD47 on the surface of tumor cells enables them to evade phagocytosis, thereby facilitating tumor growth and progression [19,20,21,22]. This phenomenon has been correlated with poor survival outcomes in studies of gastric and ovarian cancer [23,24]. Moreover, various approaches, such as monoclonal antibodies that block CD47-SIRPα interaction, appear to have promising and innovative potential in cancer immunotherapy [25,26]. Research on lung and breast cancers has shown that antibody–drug conjugates (ADCs) developed against CD47 can inhibit tumor growth and eliminate radioresistant cells [27,28,29]. Despite these efforts, challenges such as managing side effects and overcoming resistance mechanisms remain [30].

CD68, also known as macrosialin, is a transmembrane glycoprotein found in lysosomes and late endosomes of cells belonging to the mononuclear phagocytic system, particularly macrophages, monocytes, and dendritic cells [31]. It plays a role in various cellular functions, including phagocytosis, endocytosis, antigen presentation, and lysosomal degradation. Specifically, it facilitates the uptake and digestion (phagocytosis) of foreign particles, cellular debris, and pathogens by macrophages, thereby contributing to tissue homeostasis and immune defense. Under inflammatory conditions, CD68 expression typically increases in activated macrophages and infiltrating monocytes, leading to enhanced phagocytic activity [31]. Consequently, in tissue immunohistochemical analyses, CD68 is widely used as a marker for macrophages and monocytes. Increased expression of CD68 in tumor tissues has been associated with favorable survival outcomes in head and neck cancers [32]. However, in many other cancer types such as glioblastoma, pancreas, liver, lung, kidney, and thyroid cancers, it has been linked to tumor progression, metastasis, and poor clinical outcomes [33,34]. Moreover, based on findings from studies across different cancer types [35,36], its interaction with receptors directly influences immune checkpoint pathways, such as PD-1 and PD-L1, and its potential impact on resistance mechanisms suggest that it is a promising therapeutic target in cancer immunotherapy.

CD163 is a transmembrane glycoprotein belonging to the scavenger receptor cysteine-rich (SRCR) superfamily and is expressed on the surface of monocyte–macrophage lineage cells [37]. It plays a crucial role in maintaining iron homeostasis and protecting tissues from oxidative stress by interacting with the haptoglobin–hemoglobin complex to facilitate the clearance of free hemoglobin and prevent oxidative damage [37]. Additionally, CD163 can trigger anti-inflammatory signaling pathways within macrophages, leading to the production of anti-inflammatory cytokines, such as interleukin-10, and suppression of pro-inflammatory responses [37]. Increased CD163-positive tumor-associated macrophages (TAMs) in the tumor microenvironment have been associated with tumor progression, metastasis, and poor prognosis in specific cancer types [38,39,40,41,42,43]. Recent research, particularly focusing on immunotherapy, has shown promising potential for developing new therapeutic approaches and improving treatment options.

The primary objective of this study was to evaluate the prognostic significance of CD47, CD68, and CD163 expression levels, identified as TAM biomarkers, on the survival outcomes of patients with stage II-IVB NPC. Moreover, this study examined the potential influence of prognostic indices, such as the monocyte-to-lymphocyte ratio (MLR) and the monocyte-to-albumin ratio (MAR), which are evaluated prior to treatment and predominantly reflect monocyte activity in peripheral blood, on therapeutic efficacy and clinical outcomes in NPC. Additionally, the correlation between these indices and TAMs, as determined through immunohistochemical analysis of tumor tissues, has been explored. To the best of our knowledge, this study is the first to research to simultaneously investigate TAM biomarkers (CD47, CD68, and CD163) in a triple combination in NPC. Furthermore, the current study holds promise in the rapidly accelerating field of research focused on immune checkpoint pathways, suggesting potential for the development of new combinations, overcoming resistance mechanisms, and expanding treatment options in near the future.

## 2. Materials and Methods

### 2.1. Study Design, Patient Selection, and Collection of the Data

**Figure 1:**
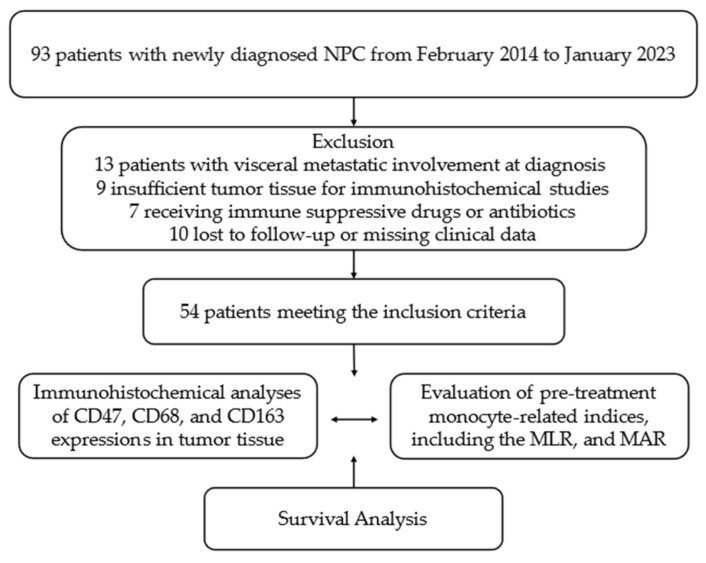
Flowchart of the study according to CONSORT diagram. Abbreviations: NPC, nasopharyngeal carcinoma; MLR, monocyte-to-lymphocyte ratio; MAR, monocyte-to-albumin ratio; CONSORT, consolidated standards of reporting trials.

Following ethical approval from the Institutional Ethics Committee (approval number: 2023-342), 93 patients treated at the Clinical Oncology Department of Health Sciences University Antalya Education and Research Hospital (HSUAERH) with a pathologically confirmed diagnosis of NPC between February 2014 and May 2023 were retrieved from the archival records. Given the high potential for long-lasting responses to local treatments in stage 1 nasopharyngeal cancer, with 5-year survival rates of 85–90%, early-stage patients were excluded from the study to mitigate challenges in interpreting disease-free and overall survival data during clinical follow-up. Due to the substantial disparity in 5-year survival rates between patients with distant visceral organ involvement (10–30%) and those with locally advanced or oligometastatic disease (60–75%), 13 patients presenting with visceral metastatic involvement at the time of diagnosis were excluded from the study. Additionally, nine patients with inadequate tumor tissue for immunohistochemical analysis of CD47, CD68, and CD163 were excluded. Patients younger than 18 years or older than 80 years, those with an uncontrolled second primary cancer diagnosis, seven patients with a history of prolonged immunosuppressive treatment due to chronic immune or inflammatory conditions or antibiotic use, and ten patients with incomplete medical laboratory or radiological data during clinical follow-up were excluded from the study. In conclusion, 54 patients who adhered to the core design and met all the study criteria were included in the final analysis (Figure 1).

The medical records of the patients, including demographic and clinical data such as age, sex, Eastern Cooperative Oncology Group (ECOG) performance status, body mass index, smoking status, presence of comorbidities, alcoholism, pre-treatment peripheral complete blood count, comprehensive biochemical analyses, plasma EBV-DNA copy number, tumor node metastasis (TNM) category, tumor grade, clinical stage, disease burden categorized as locoregional or oligometastatic, induction chemotherapy status, chosen induction chemotherapy regimen, response after induction chemotherapy, chemotherapy agents administered with CCRT, response after CCRT, adjuvant chemotherapy status, chosen adjuvant chemotherapy regimen, and development of progression and mortality during clinical follow-up, were obtained from the archives of the oncology department at HSUAERH. MLR was calculated using the formula described by Xiang et al. [44]: MLR = (monocytes/lymphocytes). MAR was calculated according to Zhao et al. [45]: MAR = (monocyte/albumin).

### 2.2. Treatment Details and Response Assessment

All patients included in the study were staged according to the American Joint Committee on Cancer (AJCC) 8th edition, utilizing Fluorine-18 fluorodeoxyglucose (FDG) positron emission tomography (PET) and magnetic resonance imaging (MRI) scans prior to treatment initiation. For patients classified as stage II to IVb, the standard treatment protocol comprised either CCRT or induction chemotherapy with cisplatin–gemcitabine or cisplatin–5FU followed by CCRT [12]. All patients were administered cisplatin or carboplatin as a part of CCRT. Subsequently, patients under clinical follow-up were monitored every three months through serial radiological examinations and blood tests. Clinical responses were assessed and categorized as complete response (CR), partial response (PR), stable disease (SD), or progressive disease (PD) in accordance with the revised Response Evaluation Criteria in Solid Tumors (RECIST) guidelines (version 1.1). Progression-free survival (PFS) was defined as the time from the date of initial diagnosis to progression, death, or the last follow-up. Overall survival (OS) was calculated as the time from the date of histological diagnosis to death or last follow-up. The primary endpoint of interest was the OS.

### 2.3. Immunohistochemical Study and CD47-CD68-CD163 Scoring System

Tissue sections were acquired from slides that had been pre-coated and cut from tissue blocks at a thickness of 4 μm. Deparaffinization was performed using xylene followed by rehydration with graded ethanol solutions. The tissue sections were then incubated overnight at 4 °C in a humidified chamber with rabbit recombinant monoclonal antibody CD47 (EPR21794 CN), mouse recombinant monoclonal antibody CD68 (KP1 CN), and rabbit recombinant monoclonal antibody CD163 (EPR19518 CN) at dilution ratios of 1:750, 1:1000, and 1:500, respectively (Abcam, Cambridge, United Kingdom) (CN;clone number). Ultimately, two head and neck pathologists blinded to the clinical data assessed and scored the stained tissue slides.

Prostate tissue was utilized as the positive control for CD47, while tonsil tissue served as the positive control for CD68 and CD163. The slides stained for CD47, CD68, and CD163 were examined under a light microscope. Membranous or cytoplasmic staining was considered positive for CD47, whereas dot-like granular or cytoplasmic staining was regarded as positive for CD68 and CD163.

**Figure 2:**
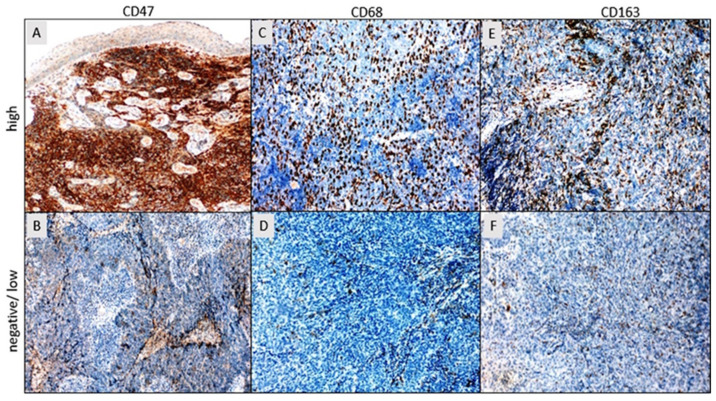
(**A**) High expression of CD47 in tumor tissue. (**B**) Negative expression of CD47 in tumor tissue. (**C**) High expression of CD68 in peritumoral and intratumoral macrophages. (**D**) Negative expression of CD68 in peritumoral and intratumoral macrophages. (**E**) High expression of CD163 in peritumoral and intratumoral macrophages. (**F**) Low expression of CD163 in peritumoral and intratumoral macrophages (DAB, ×100). DAB (diaminobenzidine).

The staining intensity was categorized according to the following scale: 0, absence of staining; 1, weak staining; 2, moderate staining; and 3, strong staining (Figure 2). Tumor cell staining percentage was categorized based on the following criteria: 0 for staining less than 10% (considered negative), 1 for staining 10% or more but less than 50%, 2 for staining 50% or more but less than 80%, and 3 for staining 80% or more [46]. The final scores for CD47, CD68, and CD163 were determined by summing the stain density (rated from 0 to 3) and stain coverage percentage (rated from 0 to 3), yielding a combined score ranging from a minimum of 0 to a maximum of 6 for each case. Patients were categorized into two distinct groups according to their CD47, CD68, and CD163 cumulative expression scores: negative/low (0–2/3 points) and high (4–6 points) (Figure 2). Subsequent comparative statistical analyses of the clinical data were performed using this classification scheme.

Ethical considerations were adhered to throughout this study, which was conducted in compliance with the Helsinki Declaration of 1964 as revised in 2013. The study protocol was thoroughly reviewed and approved by the institutional review board of the HSUAERH (Approval Number: 2023-342). Given the retrospective design of this study, patient consent was not mandatory. Nonetheless, to safeguard patient confidentiality, data were anonymized.

### 2.4. Statistical Analysis

Statistical analyses were performed using Statistical Package for Social Sciences (SPSS) version 27 for Windows (IBM SPSS Inc., Chicago, IL, USA). The sample size was calculated using the G-Power 3.1.9.2 program. With an effect size of 0.40, a type I error rate of 0.05, and a test power of 95%, it was determined that a sample size of 52 tissue samples would be sufficient for the study. The normal distribution suitability of continuous data was evaluated using the Kolmogorov–Smirnov and Shapiro–Wilk tests. Numerical variables conforming to a normal distribution are expressed as mean ± standard deviation, whereas those deviating from normality are presented as median (min–max). The predictive accuracy of MLR and MAR for mortality was evaluated using receiver operating characteristic (ROC) curve analysis. The optimal cut-off values for the MLR and MAR ratios were determined using the Youden Index method within the ROC curve analysis. Continuous data were compared using the independent samples *t*-test or Mann–Whitney U test. Categorical data were compared using Pearson’s chi-squared test. Fisher’s exact test was used when expected value problems arose. PFS and OS were estimated using the Kaplan–Meier method and compared using the log-rank test. Variables significantly associated with survival in univariate analysis were further analyzed using multivariate Cox regression models. Statistical significance was defined as *p* < 0.05 for all analyses.

## 3. Results

The median age of the cohort was 57 years (range: 19–82 years). Thirty-five patients (64.8%) were aged > 55 years, and 43 patients (79.6%) were male. A history of smoking was documented in 34 patients (63%), whereas 13 patients (24.1%) reported a history of alcohol consumption. Seventeen patients had comorbid conditions, predominantly cardiovascular disease. EBV-DNA levels exceeding 65 copies/mL were detected in 32 patients (59.3%). The ECOG PS was 0–1 in 49 patients and 2 in 5 patients. In the majority of patients (64.8%), the tumor grade was 3. According to TNM staging, 48.1% of the patients had T3-4 tumors, and 59% had N2-3 lymph node involvement. According to clinical staging, 32 patients (59.3%) were classified as having Stage II-III disease, whereas 22 patients (40.7%) were classified as having Stage IVA-IVB disease. At diagnosis, 46 patients (85.2%) presented with locoregional disease, while 8 patients (14.8%) had oligometastatic disease. Among the 46 patients (%85.2) who received induction chemotherapy, 28 (60.9%) were treated with cisplatin plus gemcitabine, while 18 (39.1%) received a modified DCF regimen. The response to induction chemotherapy was assessed based on the RECIST criteria. SD was observed in 15 patients (32.6%), PR in 21 (45.7%), and CR in 10 (21.7%). All patients underwent CCRT with either cisplatin (87%) or carboplatin (13%). In the response evaluation following CCRT, seven patients exhibited PD, 15 patients had SD, and 32 patients showed either PR or CR. Adjuvant chemotherapy was administered to 37 (68.5%) patients. The most frequently used regimen was cisplatin plus fluorouracil (5FU), administered to 22 patients, followed by gemcitabine in 9 patients, and capecitabine in 6 patients. Table 1 provides a detailed summary of the sociodemographic and clinical characteristics of NPC patients categorized according to CD47, CD68, and CD163 expression levels.

### 3.1. Cut-Off Values of the Laboratory Parameters

**Figure 3:**
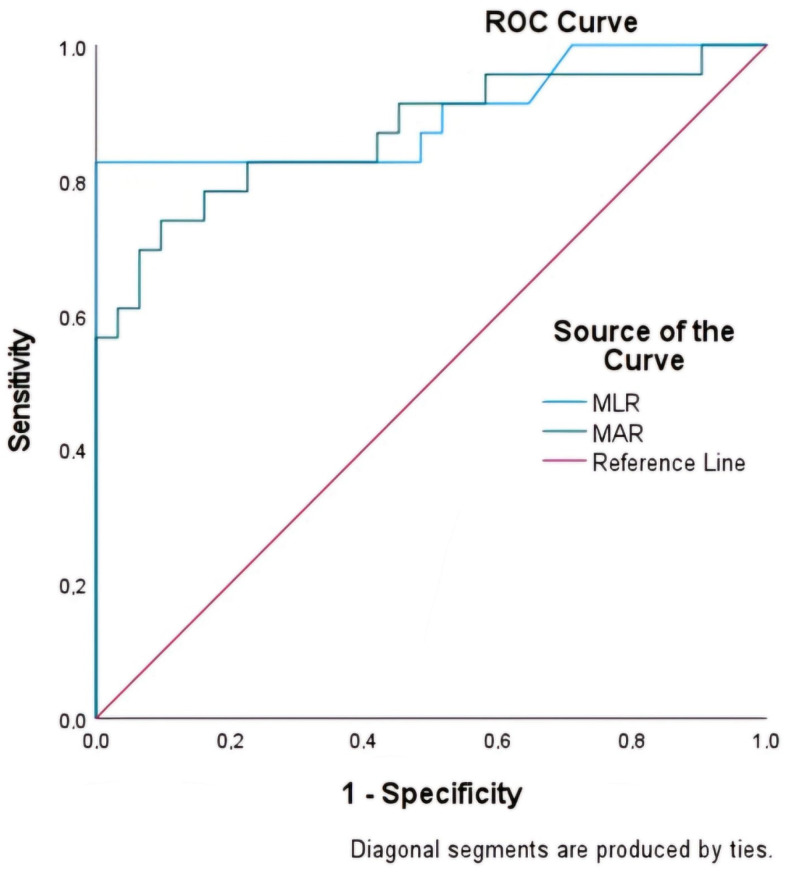
Comparison of the capability of MLR and MAR to predict mortality in nasopharyngeal carcinoma using ROC curve analysis.

The MLR and MAR indices were assessed for their predictive efficacy with respect to mortality using ROC curve analysis (Table 2). MLR exhibited the highest area under the ROC curve (AUC) at 0.898 (95% CI: 0.80–0.99), followed by MAR at 0.870 (95% CI: 0.76–0.97) (Figure 3). The optimal cutoff values, determined using the maximum Youden index, were 0.545 for MLR and 16.145 for MAR.

CD47 expression was negative or low in 29 (53.7%) NPC patients, whereas 25 (46.3%) had high CD47 expression. High CD47 expression was significantly associated with older age (*p* = 0.029), lower ECOG PS (*p* = 0.017), elevated EBV-DNA levels (*p* = 0.004), advanced clinical stage (*p* = 0.008), oligometastatic burden (*p* = 0.001), reduced response to CCRT (*p* < 0.001), increased MLR and MAR (*p* < 0.001), and higher CD68 and CD163 expressions (*p* < 0.001) (Table 1 and Table 3).

CD68 expression was negative or low in 25 (46.3%) NPC patients, whereas 29 (53.7%) had high CD68 expression. High CD68 expression was significantly associated with advanced clinical stage (*p* = 0.004), oligometastatic burden (*p* = 0.004), reduced response to CCRT (*p* < 0.001), increased MLR (*p* < 0.001), increased MAR (*p* = 0.006), and higher CD47 and CD163 expressions (*p* < 0.001) (Table 1 and Table 3).

CD163 expression was negative or low in 23 (42.6%) NPC patients, whereas 31 (57.4%) had high CD163 expression. High CD163 expression was significantly associated with older age (*p* = 0.025), elevated EBV-DNA levels (*p* = 0.04), oligometastatic burden (*p* = 0.008), reduced response to CCRT (*p* = 0.005), increased MLR and MAR (*p* < 0.001), and higher CD47 and CD68 expressions (*p* < 0.001) (Table 1 and Table 3). The results of the comparison of the CD47, CD68, and CD163 expression levels are presented in Table 3.

### 3.2. Survival Analysis

**Figure 4:**
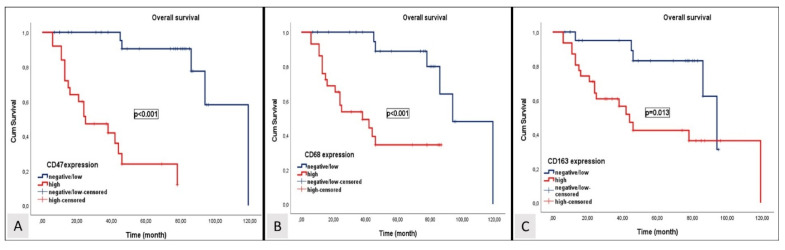
Kaplan–Meier curves depicting overall survival stratified by CD47 (**A**), CD68 (**B**), and CD163 (**C**) expression levels.

**Figure 5:**
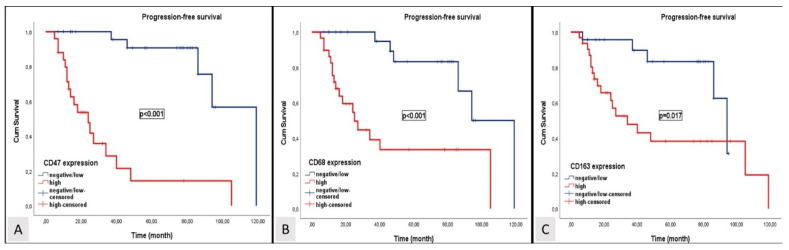
Kaplan–Meier curves depicting progression-free survival stratified by CD47 (**A**), CD68 (**B**), and CD163 (**C**) expression levels.

Over a median follow-up period of 47.2 months, disease progression was observed in 25 patients (46.3%), and 19 patients (35.1%) died. In patients with locally advanced and oligometastatic NPC, the median OS and PFS were 86 months (95% CI; 46.3–125.7) and 77 months (95% CI; 35.5- 118.5). The median OS levels for patients exhibiting high expression levels of CD47, CD68, and CD163 were 25 months (95% CI; 6.3–43.6), 38 months (95% CI; 12.1–63.9), and 44 months (95% CI; 33.1–54.9), respectively. The median OS levels for patients with negative or low expression levels of CD47, CD68, and CD163 were 109 months (95% CI; 88.2–120.2), 94 months (95% CI: 75.1–112.9), and 94 months (95% CI: 81.9–106.1). The median PFS levels for patients exhibiting high expression levels of CD47, CD68, and CD163 were 21 months (95% CI; 10.4–31.6), 25 months (95% CI; 12.9–37.1), and 34 months (95% CI; 12.8–55.2), respectively. The median PFS levels for patients with negative or low expression levels of CD47, CD68, and CD163 were 87 months (95% CI; 66.9–107.4), 85 months (95% CI: 50.6–118.5), and 79 months (95% CI: 61.9–96.1). Patients with low or negative expression levels of CD47, CD68, and CD163 exhibited significantly longer OS and PFS than those with high expression levels of CD47, CD68, and CD163. Kaplan–Meier survival curves for OS and PFS stratified by negative or low and high expression levels of CD47, CD68, and CD163 are shown in Figure 4 and Figure 5, respectively.

The clinical and immunohistochemical data affecting the OS of patients with NPC were investigated using a univariate Cox proportional hazards model (Table 4). In univariate analysis, age, alcohol consumption, EBV-DNA level, clinical stage, disease burden, MLR, MAR, CD47, CD68, and CD163 were significantly associated with OS (*p* < 0.05). In the multivariate analysis, MLR, MAR, and CD163 were significantly associated with overall survival (*p* < 0.05) (Table 4). In the univariate Cox proportional hazards model, the following factors were significantly associated with PFS (*p* < 0.05): age, alcohol consumption, EBV-DNA level, clinical stage, MLR, MAR, CD47, CD68, and CD163 (Table 5). In multivariate analysis, only MLR demonstrated a statistically significant association with PFS (*p* < 0.05) (Table 5). Both univariate and multivariate analyses revealed that high expression levels of CD163 or high MLR and MAR scores were adverse prognostic factors associated with reduced OS (Table 4). An elevated MLR score independently constituted a risk factor for both OS and PFS in patients with NPC (Table 4 and Table 5). Furthermore, it was a robust predictor of adverse clinical outcomes.

## 4. Discussion

TAMs are key components of the tumor microenvironment and play a crucial role in cancer progression, immune evasion, and metastasis. TAMs are typically polarized into a phenotype that supports tumor growth, often referred to as the M2-like phenotype. The expression of specific biomarkers such as CD47, CD68, and CD163 is associated with these TAMs and has been studied extensively in relation to cancer prognosis [46,47,48,49,50]. Elevated expression levels of CD47, CD68, and CD163 are commonly associated with a more immunosuppressive tumor microenvironment, which promotes tumor growth, metastasis, and resistance to treatment. Extensive cancer research has shown that tumors with high expression of these biomarkers are associated with poorer prognosis, characterized by reduced OS and PFS [46,48,49,50]. This prompted the investigation of therapeutic strategies aimed at reprogramming or inhibiting TAMs. For example, targeting CD47 with monoclonal antibodies has been explored in clinical trials, demonstrating potential benefits beyond its direct antitumor effects, such as enhancing the efficacy of conventional treatments such as chemotherapy and immunotherapy and addressing resistance to radiotherapy [26,27,28,30]. Thus, CD47, CD68, and CD163 not only serve as prognostic indicators but also represent promising targets for novel cancer therapies designed to modulate the tumor immune microenvironment. In addition, peripheral monocytes play a pivotal role in the formation and function of TAMs within tumors. Recruitment and differentiation are critical processes that influence tumor progression and prognosis. By understanding these mechanisms, researchers are developing strategies to modulate TAMs activity and to improve cancer treatment outcomes.

The findings of this study provide compelling evidence that elevated expression levels of CD47, CD68, and CD163 in tumor tissues are significantly correlated with poorer PFS and OS in patients with NPC. The higher incidence of adverse prognostic factors, such as oligometastatic burden (advanced clinical stage), diminished response to CCRT, and increased MLR and MAR scores in patients with high CD47, CD68, and CD163 expression, underscores the prognostic relevance of TAMs. Regression analyses further revealed that high CD163 expression and elevated MLR scores exerted a more pronounced predictive influence on clinical outcomes. This finding suggests that, in addition to CD47, which has been extensively investigated in tumor immunology and antibody–drug conjugate research, CD163 may also represent a novel target for future immunotherapeutic strategies. Moreover, this study highlights that, beyond TAMs, readily calculable and accessible prognostic indices, such as MLR and MAR, which reflect peripheral blood monocyte activity, could offer valuable prognostic insights to clinicians, aiding in the prediction of clinical outcomes and guiding therapeutic decision making, particularly in cancers such as NPC. In conclusion, this study contributes a novel perspective to the field of tumor immunology, providing promising avenues for future clinical research aimed at expanding treatment options, overcoming resistance mechanisms, and improving clinical outcomes.

The expression of specific TAMs, such as CD47, CD68, and CD163, has been studied in NPC and is linked to various aspects of tumor behavior, prognosis, and potential therapeutic strategies. In a study by Wang et al. [51], increased CD47 expression levels correlated with higher recurrence rates and elevated EBV-DNA levels in non-metastatic NPC, mirroring the findings of our research. A study by Yu et al. [52] suggested that elevated CD68 expression might predict better prognostic outcomes in patients with NPC. While these findings appear contradictory to our study’s data, differences in the study period, the heterogeneity of the selected patient population, and the fact that the majority of the cohort received radiotherapy alone as standard treatment are factors that may influence the interpretation of the results. A meta-analysis by Chen et al. [53] on NPC prognosis, which included research on mouse and human models, the density of the M2-like TAM markers CD68 and CD163 was associated with poorer overall survival. Yu et al. [54] also found that increased CD163 expression levels were linked to worsened OS and PFS outcomes in NPC, consistent with the results of our study, given its similar design and patient distribution. In a study by Deng et al. [55], which focused on immunotherapy in NPC, it was found that higher PDL-1 expression levels correlated with lower CD163 expression, and both were associated with more favorable survival outcomes. The findings of this study also support the notion that CD163 could be a critical target molecule in treatment combinations involving immunotherapy for advanced NPC.

Our findings are consistent with existing molecular studies on the prognosis and clinical trajectory of nasopharyngeal cancer. Additionally, the notion that indices based on immune inflammation and nutrition, such as MLR and MAR, which are indirectly associated with TAMs activity, can further contribute to risk stratification among patients and prediction of the clinical course of cancer is supported. This study further corroborates that established risk factors, including advanced age, poor ECOG PS, and elevated EBV-DNA copy number, adversely affect disease progression. While these TAM-associated biomolecules (CD47, CD68, and CD163) negatively affect overall survival in NPC, CD163 has emerged as a particularly significant biomarker due to its exceptional predictive value, suggesting its potential as a promising target in clinical research involving ADCs. This study offers a novel perspective for future research focused on developing alternative therapeutic strategies, addressing resistance mechanisms, and broadening the treatment options for NPC.

The strength and adequacy of this study are underscored by several consistent and positive factors, including the inclusion of a substantial number of cases within a relatively rare tumor group and the treatment of all patients according to international standard protocols by a tertiary cancer center with an active multidisciplinary tumor board. However, this study has some limitations. The retrospective design, small sample size, and single-center nature of the study may limit the balanced distribution of cases, the application of more comprehensive statistical analyses, and the generalizability of the findings, potentially impacting the robustness of the study. Additionally, the potential for bias due to differences in adjuvant and advanced-line treatment options, which could influence overall survival outcomes, should be considered. The inclusion of regional and ethnic variations, particularly for endemic cancer, may further complicate the interpretation of clinical outcomes. Moreover, the absence of a globally recognized and standardized protocol for immunohistochemical evaluation of CD47, CD68, and CD163 could result in variability in the analysis. However, regarding the calculation of prognostic indices such as MLR and MAR, certain issues may have been overlooked, including the presence of asymptomatic infections during the measurement of blood parameters, individual differences in immune system alterations, potential transient fluctuations in marker levels, and the lack of an internal validation group.

## 5. Conclusions

This study showed that elevated expression levels of CD47, CD68, and CD163 in tumor tissues are significantly correlated with poorer OS and PFS in patients with NPC. Increased levels of these biomarkers, together with higher MLR and MAR, reflect a more immunosuppressive tumor microenvironment, which is associated with advanced disease stage, diminished response to CCRT, and unfavorable clinical outcomes. These findings underscore the potential of CD47, CD68, and CD163 as robust prognostic indicators and their promise as targets for novel therapeutic strategies aimed at modulating the tumor immune environment. Additionally, the substantial predictive value of MLR and MAR reinforces their role as accessible and valuable prognostic tools in the management of NPC. Future research should focus on validating these results in larger, more diverse patient cohorts and exploring the development of targeted therapies that combine the inhibition of these biomarkers with existing treatment modalities. This study provides critical insights into NPC prognosis and proposes promising pathways to enhance therapeutic strategies and improve patient outcomes.

## Figures and Tables

**Table 1:** Comparison of sociodemographic and clinicopathological characteristics of patients classified according to CD47, CD68, and CD163 expression levels (all patients, *n* = 54).

Variables		CD47 Expression			CD68 Expression			CD163 Expression		
		Negative/Low	High	*p* *	Negative/Low	High	*p* *	Negative/Low	high	*p* *
		*n*, (%)	*n*, (%)		*n*, (%)	n, (%)		*n*, (%)	*n*, (%)	
Age	<55	14 (48.3)	5 (20)	0.029	10 (40)	9 (31)	0.343	12 (52.2)	7 (22.6)	0.025
	≥55	15 (51.7)	20 (80)		15 (60)	20 (69)		11 (47.8)	24 (77.4)	
Sex	Male	21 (72.4)	22 (88)	0.14	20 (80)	23 (79.3)	0.61	19 (82.6)	24 (77.4)	0.454
	Female	8 (27.6)	3 (12)		5 (68)	6 (20.7)		4 (17.4)	7 (22.6)	
ECOG PS	0–1	29 (100)	20 (80)	0.017	24 (96)	25 (86.2)	0.225	22 (95.7)	27 (87.1)	0.283
	2	0 (0)	5 (20)		1 (4)	4 (13.8)		1 (4.3)	4 (12.9)	
Smoking status	No	10 (34.5)	10 (40)	0.445	9 (36)	11 (37.9)	0.555	7 (30.4)	13 (41.9)	0.282
	Yes	19 (65.5)	15 (60)		16 (64)	18 (62.1)		16 (69.6)	18 (58.1)	
Comorbidity	No	21 (72.4)	16 (64)	0.355	17 (68)	20 (69)	0.585	18 (78.3)	19 (61.3)	0.151
	Yes	8 (27.6)	9 (36)		8 (32)	9 (31)		5 (21.7)	12 (38.7)	
Alcohol consumption	No	21 (72.4)	20 (80)	0.372	19 (76)	22 (75.9)	0.622	17 (73.9)	24 (77.4)	0.506
	Yes	8 (27.6)	5 (20)		6 (24)	7 (24.1)		6 (26.1)	7 (22.6)	
EBV-DNA copy number	Low	17 (53.1)	5 (20)	0.004	13 (52)	9 (31)	0.099	13 (56.5)	9 (29)	0.04
	High	12 (46.9)	20 (80)		12 (48)	20 (69)		10 (43.5)	22 (71)	
Clinical stage	II-III	22 (75.9)	10 (40)	0.008	20 (80)	12 (41.4)	0.004	17 (73.9)	15 (48.4)	0.053
	IVA-IVB	7 (24.1)	15 (60)		5 (20)	17 (58.6)		6 (26.1)	16 (51.6)	
Disease burden	Locoregional	29 (100)	17 (68)	0.001	25 (100)	21 (72.4)	0.004	23 (100)	23 (74.2)	0.008
	Oligometastatic	0 (0)	8 (32)		0 (0)	8 (27.6)		0 (0)	8 (25.8)	
Response to CCRT	PD	1 (3.4)	6 (24)	<0.001	1 (4)	6 (20.7)	<0.001	1 (4.3)	6 (19.4)	0.005
	SD	3 (10.4)	12 (48)		1 (4)	14 (48.3)		3 (13.1)	12 (38.7)	
	PR + CR	25 (86.2)	7 (28)		23 (92)	9 (31)		19 (82.6)	13 (41.9)	
MLR	Low	28 (96.6)	7 (28)	<0.001	23 (92)	12 (41.4)	<0.001	21 (91.3)	14 (45.2)	<0.001
	High	1 (3.4)	18 (72)		2 (8)	17 (58.6)		2 (8.7)	17 (54.8)	
MAR	Low	27 (93.1)	8 (32)	<0.001	21 (84)	14 (48.3)	0.006	22 (95.7)	13 (41.9)	<0.001
	High	2 (6.1)	17 (68)		4 (16)	15 (51.7)		1 (4.3)	18 (58.1)	

Abbreviations: ECOG PS, Eastern Cooperative Oncology Group Performance Status; EBV, Epstein–Barr virus (≥65 copies/mL = positive or high); CCRT, concurrent chemoradiotherapy; PD, progressive disease; SD, stable disease; PR, partial remission; CR, complete remission; MLR, monocyte-to-lymphocyte ratio; MAR, monocyte-to-albumin ratio; * statistically significant (*p* <0.05).

**Table 2:** AUC values for MLR and MAR compared using ROC curve analysis.

	AUC	95% CI	Sensitivity	Specificity	*p*
MLR	0.898	0.800–0.995	82.6%	100%	<0.0001
MAR	0.870	0.766–0.973	82.6%	77.4%	<0.0001

Abbreviations: AUC, area under the curve; CI, confidence interval; MLR, monocyte-to-lymhocyte ratio; MAR, monocyte-to-albumin ratio.

**Table 3:** The relationship between the expressions of CD47, CD68, and CD163.

		CD47		*p*			CD68		*p*			CD163		*p*
		Negative/Low	High				Negative/Low	High				Negative/Low	High	
CD68	0/low	23 (79.3)	2 (8.0)	<0.001	CD163	0/low	18 (72.0)	5 (17.2)	<0.001	CD47	0/low	20 (87.0)	9 (29.0)	<0.001
	high	6 (20.7)	23 (92.0)			high	7 (28.0)	24 (82.8)			high	3 (13.0)	22 (71.0)	
CD163	0/low	20 (69.0)	3 (12.0)	<0.001	CD47	0/low	23 (92.0)	6 (20.7)	<0.001	CD68	0/low	18 (78.3)	7 (22.6)	<0.001
	high	9 (31.0)	22 (88.0)			high	2 (8.0)	23 (79.3)			high	5 (21.7)	24 (77.4)	

**Table 4:** Cox regression analysis of overall survival in patients with locally advanced and oligometastatic nasopharyngeal carcinoma.

Overall Survival							
	Univariate Analysis				Multivariate Analysis		
	HR	95% CI	*p*		HR	95% CI	*p*
Age	0.07	0.01–0.54	0.011	Age	0.17	0.02–1.54	0.115
Sex	0.51	0.17–1.53	0.228	Sex	-	-	-
Smoking	1.47	0.60–3.57	0.398	Smoking	-	-	-
Alcohol	0.12	0.02–0.86	0.035	Alcohol	0.61	0.07–5.28	0.652
EBV-DNA	3.08	1.43–10.10	0.007	EBV-DNA	5.17	0.94–28.54	0.059
ECOG PS	3.01	1.00–9.50	0.050	ECOG PS	1.49	0.35–6.35	0.587
Clinical stage	2.80	1.17–6.71	0.021	Clinical stage	0.59	0.22–1.59	0.29
Disease burden	4.40	1.63–11.87	0.003	Disease burden	2.74	0.65–11.48	0.169
MLR	84.93	11.06–652.3	<0.001	MLR	187.14	6.79–5151.0	0.002
MAR	11.34	4.06–31.81	<0.001	MAR	9.76	1.50–63.45	0.017
CD47	19.84	4.50–87.57	<0.001	CD47	0.33	0.01–16.36	0.574
CD68	5.79	1.92–17.42	0.002	CD68	8.82	0.42–184.03	0.160
CD163	3.31	1.21–9.06	0.019	CD163	0.34	0.03–0.45	0.010

Abbreviations: EBV, Epstein–Barr virus; ECOG PS, Eastern Cooperative Oncology Group Performance Status; MLR, monocyte-to-lymphocyte ratio; MAR, monocyte-to-albumin ratio.

**Table 5:** Cox regression analysis of progression-free survival in patients with locally advanced and oligometastatic nasopharyngeal carcinoma.

Progression-Free Survival							
	Univariate Analysis				Multivariate Analysis		
	HR	95% CI	*p*		HR	95% CI	*p*
Age	0.87	0.01–0.65	0.017	Age	0.31	0.03–2.84	0.299
Sex	0.51	0.17–1.51	0.223	Sex	-	-	-
Smoking	1.30	0.54–3.12	0.558	Smoking	-	-	-
Alcohol	0.13	0.02–0.96	0.046	Alcohol	0.49	0.04–5.57	0.566
EBV-DNA	3.32	1.28–8.62	0.014	EBV-DNA	1.05	0.26–4.28	0.941
ECOG PS	2.89	0.96–8.72	0.060	ECOG PS	-	-	-
Clinical stage	2.66	1.10–6.40	0.029	Clinical stage	0.79	0.28–2.30	0.671
Disease burden	2.31	0.88–6.12	0.091	Disease burden	-	-	-
MLR	22.65	16.53–78.64	<0.001	MLR	14.77	1.92–113.45	0.010
MAR	7.68	2.95–20.03	<0.001	MAR	1.50	0.29–7.69	0.625
CD47	11.80	3.86–36.10	<0.001	CD47	1.85	0.12–28.78	0.574
CD68	5.39	1.95–14.90	0.001	CD68	2.52	0.30–20.95	0.394
CD163	3.21	1.17–8.84	0.024	CD163	0.14	0.02–1.33	0.087

Abbreviations: EBV, Epstein–Barr virus; ECOG PS, Eastern Cooperative Oncology Group Performance Status; MLR, monocyte-to-lymphocyte ratio; MAR, monocyte-to-albumin ratio.

## Data Availability

The datasets used in this study can be made available by the corresponding author upon reasonable request, with permission from the Clinical Oncology Department of HSUAERH.

## References

[1] Sung H., Ferlay J., Siegel R.L., Laversanne M., Soerjomataram I., Jemal A., Bray F. (2021). Global Cancer Statistics 2020: GLOBOCAN Estimates of Incidence and Mortality Worldwide for 36 Cancers in 185 Countries. CA Cancer J. Clin..

[2] Chang E.T., Ye W., Zeng Y.-X., Adami H.-O. (2021). The Evolving Epidemiology of Nasopharyngeal Carcinoma. Cancer Epidemiol. Biomark. Prev..

[3] Zhang Y., Rumgay H., Li M., Cao S., Chen W. (2023). Nasopharyngeal Cancer Incidence and Mortality in 185 Countries in 2020 and the Projected Burden in 2040: Population-Based Global Epidemiological Profiling. JMIR Public Health Surveill..

[4] Su Z.Y., Siak P.Y., Lwin Y.Y., Cheah S.-C. (2024). Epidemiology of Nasopharyngeal Carcinoma: Current Insights and Future Outlook. Cancer Metastasis Rev..

[5] London A.O., Gallagher L.W., Sharma R.K., Spielman D., Golub J.S., Overdevest J.B., Yan C.H., DeConde A., Gudis D.A. (2022). Impact of Race, Ethnicity, and Socioeconomic Status on Nasopharyngeal Carcinoma Disease-Specific and Conditional Survival. J. Neurol. Surg. B Skull Base.

[6] Luo W. (2023). Nasopharyngeal Carcinoma Ecology Theory: Cancer as Multidimensional Spatiotemporal “Unity of Ecology and Evolution” Pathological Ecosystem. Theranostics.

[7] Chen Y.-P., Chan A.T.C., Le Q.-T., Blanchard P., Sun Y., Ma J. (2019). Nasopharyngeal Carcinoma. Lancet.

[8] Wong K.C.W., Hui E.P., Lo K.-W., Lam W.K.J., Johnson D., Li L., Tao Q., Chan K.C.A., To K.-F., King A.D. (2021). Nasopharyngeal Carcinoma: An Evolving Paradigm. Nat. Rev. Clin. Oncol..

[9] Jiromaru R., Nakagawa T., Yasumatsu R. (2022). Advanced Nasopharyngeal Carcinoma: Current and Emerging Treatment Options. Cancer Manag. Res..

[10] Huang X., Chen X., Zhao C., Wang J., Wang K., Wang L., Miao J., Cao C., Jin T., Zhang Y. (2020). Adding Concurrent Chemotherapy to Intensity-Modulated Radiotherapy Does Not Improve Treatment Outcomes for Stage II Nasopharyngeal Carcinoma: A Phase 2 Multicenter Clinical Trial. Front. Oncol..

[11] Zhang Y., Chen L., Hu G.-Q., Zhang N., Zhu X.-D., Yang K.-Y., Jin F., Shi M., Chen Y.-P., Hu W.-H. (2019). Gemcitabine and Cisplatin Induction Chemotherapy in Nasopharyngeal Carcinoma. N. Engl. J. Med..

[12] Chen Y.-P., Ismaila N., Chua M.L.K., Colevas A.D., Haddad R., Huang S.H., Wee J.T.S., Whitley A.C., Yi J.-L., Yom S.S. (2021). Chemotherapy in Combination With Radiotherapy for Definitive-Intent Treatment of Stage II-IVA Nasopharyngeal Carcinoma: CSCO and ASCO Guideline. J. Clin. Oncol..

[13] Liu G.-Y., Li W.-Z., Wang D.-S., Liang H., Lv X., Ye Y.-F., Zhao C., Ke L.-R., Lv S.-H., Lu N. (2022). Effect of Capecitabine Maintenance Therapy Plus Best Supportive Care vs Best Supportive Care Alone on Progression-Free Survival Among Patients With Newly Diagnosed Metastatic Nasopharyngeal Carcinoma Who Had Received Induction Chemotherapy. JAMA Oncol..

[14] Mai H.-Q., Chen Q.-Y., Chen D., Hu C., Yang K., Wen J., Li J., Shi Y., Jin F., Xu R. (2023). Toripalimab Plus Chemotherapy for Recurrent or Metastatic Nasopharyngeal Carcinoma. JAMA.

[15] Su Z.Y., Siak P.Y., Leong C.-O., Cheah S.-C. (2022). Nasopharyngeal Carcinoma and Its Microenvironment: Past, Current, and Future Perspectives. Front. Oncol..

[16] Forder A., Stewart G.L., Telkar N., Lam W.L., Garnis C. (2022). New Insights into the Tumour Immune Microenvironment of Nasopharyngeal Carcinoma. Curr. Res. Immunol..

[17] Hayat S.M.G., Bianconi V., Pirro M., Jaafari M.R., Hatamipour M., Sahebkar A. (2020). CD47: Role in the Immune System and Application to Cancer Therapy. Cell. Oncol..

[18] Logtenberg M.E.W., Scheeren F.A., Schumacher T.N. (2020). The CD47-SIRPα Immune Checkpoint. Immunity.

[19] Huang C.-Y., Ye Z.-H., Huang M.-Y., Lu J.-J. (2020). Regulation of CD47 Expression in Cancer Cells. Transl. Oncol..

[20] Feng R., Zhao H., Xu J., Shen C. (2020). CD47: The next Checkpoint Target for Cancer Immunotherapy. Crit. Rev. Oncol. Hematol..

[21] Jiang Z., Sun H., Yu J., Tian W., Song Y. (2021). Targeting CD47 for Cancer Immunotherapy. J. Hematol. Oncol..

[22] Huang J., Liu F., Li C., Liang X., Li C., Liu Y., Yi Z., Zhang L., Fu S., Zeng Y. (2022). Role of CD47 in Tumor Immunity: A Potential Target for Combination Therapy. Sci. Rep..

[23] Shi M., Gu Y., Jin K., Fang H., Chen Y., Cao Y., Liu X., Lv K., He X., Lin C. (2021). CD47 Expression in Gastric Cancer Clinical Correlates and Association with Macrophage Infiltration. Cancer Immunol. Immunother..

[24] Yu L., Ding Y., Wan T., Deng T., Huang H., Liu J. (2021). Significance of CD47 and Its Association With Tumor Immune Microenvironment Heterogeneity in Ovarian Cancer. Front. Immunol..

[25] Liu L., Zhang L., Yang L., Li H., Li R., Yu J., Yang L., Wei F., Yan C., Sun Q. (2017). Anti-CD47 Antibody As a Targeted Therapeutic Agent for Human Lung Cancer and Cancer Stem Cells. Front. Immunol..

[26] Si Y., Zhang Y., Guan J.-S., Ngo H.G., Totoro A., Singh A.P., Chen K., Xu Y., Yang E.S., Zhou L. (2021). Anti-CD47 Monoclonal Antibody–Drug Conjugate: A Targeted Therapy to Treat Triple-Negative Breast Cancers. Vaccines.

[27] Candas-Green D., Xie B., Huang J., Fan M., Wang A., Menaa C., Zhang Y., Zhang L., Jing D., Azghadi S. (2020). Dual Blockade of CD47 and HER2 Eliminates Radioresistant Breast Cancer Cells. Nat. Commun..

[28] Chiang Z.-C., Fang S., Shen Y., Cui D., Weng H., Wang D., Zhao Y., Lin J., Chen Q. (2022). Development of Novel CD47-Specific ADCs Possessing High Potency Against Non-Small Cell Lung Cancer in Vitro and in Vivo. Front. Oncol..

[29] Nishiga Y., Drainas A.P., Baron M., Bhattacharya D., Barkal A.A., Ahrari Y., Mancusi R., Ross J.B., Takahashi N., Thomas A. (2022). Radiotherapy in Combination with CD47 Blockade Elicits a Macrophage-Mediated Abscopal Effect. Nat. Cancer.

[30] Chen Q., Guo X., Ma W. (2024). Opportunities and Challenges of CD47-Targeted Therapy in Cancer Immunotherapy. Oncol. Res..

[31] Christofides A., Strauss L., Yeo A., Cao C., Charest A., Boussiotis V.A. (2022). The Complex Role of Tumor-Infiltrating Macrophages. Nat. Immunol..

[32] Furgiuele S., Descamps G., Lechien J.R., Dequanter D., Journe F., Saussez S. (2022). Immunoscore Combining CD8, FoxP3, and CD68-Positive Cells Density and Distribution Predicts the Prognosis of Head and Neck Cancer Patients. Cells.

[33] Zhang J., Li S., Liu F., Yang K. (2022). Role of CD68 in Tumor Immunity and Prognosis Prediction in Pan-Cancer. Sci. Rep..

[34] Rezagholizadeh F., Tajik F., Talebi M., Taha S.R., Shariat Zadeh M., Farhangnia P., Hosseini H.S., Nazari A., Mollazadeh Ghomi S., Kamrani Mousavi S.M. (2024). Unraveling the Potential of CD8, CD68, and VISTA as Diagnostic and Prognostic Markers in Patients with Pancreatic Ductal Adenocarcinoma. Front. Immunol..

[35] Jiang L.-R., Zhang N., Chen S.-T., He J., Liu Y.-H., Han Y.-Q., Shi X.-Q., Yang J.-J., Mu D.-Y., Fu G.-H. (2021). PD-1-Positive Tumor-Associated Macrophages Define Poor Clinical Outcomes in Patients With Muscle Invasive Bladder Cancer Through Potential CD68/PD-1 Complex Interactions. Front. Oncol..

[36] Kato S., Okamura R., Kumaki Y., Ikeda S., Nikanjam M., Eskander R., Goodman A., Lee S., Glenn S.T., Dressman D. (2020). Expression of TIM3/VISTA Checkpoints and the CD68 Macrophage-Associated Marker Correlates with Anti-PD1/PDL1 Resistance: Implications of Immunogram Heterogeneity. Oncoimmunology.

[37] Etzerodt A., Tsalkitzi K., Maniecki M., Damsky W., Delfini M., Baudoin E., Moulin M., Bosenberg M., Graversen J.H., Auphan-Anezin N. (2019). Specific Targeting of CD163+ TAMs Mobilizes Inflammatory Monocytes and Promotes T Cell–Mediated Tumor Regression. J. Exp. Med..

[38] Xu Z., Wang L., Tian J., Man H., Li P., Shan B. (2018). High Expression of B7-H3 and CD163 in Cancer Tissues Indicates Malignant Clinicopathological Status and Poor Prognosis of Patients with Urothelial Cell Carcinoma of the Bladder. Oncol. Lett..

[39] Cheng Z., Zhang D., Gong B., Wang P., Liu F. (2017). CD163 as a Novel Target Gene of STAT3 Is a Potential Therapeutic Target for Gastric Cancer. Oncotarget.

[40] Ma C., Horlad H., Ohnishi K., Nakagawa T., Yamada S., Kitada S., Motoshima T., Kamba T., Nakayama T., Fujimoto N. (2018). CD163-Positive Cancer Cells Are Potentially Associated with High Malignant Potential in Clear Cell Renal Cell Carcinoma. Med. Mol. Morphol..

[41] Matsubara E., Komohara Y., Shinchi Y., Mito R., Fujiwara Y., Ikeda K., Shima T., Shimoda M., Kanai Y., Sakagami T. (2021). CD163-positive Cancer Cells Are a Predictor of a Worse Clinical Course in Lung Adenocarcinoma. Pathol. Int..

[42] Maisel B.A., Yi M., Peck A.R., Sun Y., Hooke J.A., Kovatich A.J., Shriver C.D., Hu H., Nevalainen M.T., Tanaka T. (2022). Spatial Metrics of Interaction between CD163-Positive Macrophages and Cancer Cells and Progression-Free Survival in Chemo-Treated Breast Cancer. Cancers.

[43] Ma S., Zhao Y., Liu X., Sun Zhang A., Zhang H., Hu G., Sun X.-F. (2022). CD163 as a Potential Biomarker in Colorectal Cancer for Tumor Microenvironment and Cancer Prognosis: A Swedish Study from Tissue Microarrays to Big Data Analyses. Cancers.

[44] Xiang J., Zhou L., Li X., Bao W., Chen T., Xi X., He Y., Wan X. (2017). Preoperative Monocyte-to-Lymphocyte Ratio in Peripheral Blood Predicts Stages, Metastasis, and Histological Grades in Patients with Ovarian Cancer. Transl. Oncol..

[45] Zhao S.-T., Chen X.-X., Yang X.-M., He S.-C., Qian F.-H. (2023). Application of Monocyte-to-Albumin Ratio and Neutrophil Percentage-to-Hemoglobin Ratio on Distinguishing Non-Small Cell Lung Cancer Patients from Healthy Subjects. Int. J. Gen. Med..

[46] Fu F., Zhang Y., Gao Z., Zhao Y., Wen Z., Han H., Li Y., Hu H., Chen H. (2021). Combination of CD47 and CD68 Expression Predicts Survival in Eastern-Asian Patients with Non-Small Cell Lung Cancer. J. Cancer Res. Clin. Oncol..

[47] Sugimura-Nagata A., Koshino A., Inoue S., Matsuo-Nagano A., Komura M., Riku M., Ito H., Inoko A., Murakami H., Ebi M. (2021). Expression and Prognostic Significance of CD47–SIRPA Macrophage Checkpoint Molecules in Colorectal Cancer. Int. J. Mol. Sci..

[48] Yuan J., He H., Chen C., Wu J., Rao J., Yan H. (2019). Combined High Expression of CD47 and CD68 Is a Novel Prognostic Factor for Breast Cancer Patients. Cancer Cell Int..

[49] Ni C., Yang L., Xu Q., Yuan H., Wang W., Xia W., Gong D., Zhang W., Yu K. (2019). CD68- and CD163-Positive Tumor Infiltrating Macrophages in Non-Metastatic Breast Cancer: A Retrospective Study and Meta-Analysis. J. Cancer.

[50] Jamiyan T., Kuroda H., Yamaguchi R., Abe A., Hayashi M. (2020). CD68- and CD163-Positive Tumor-Associated Macrophages in Triple Negative Cancer of the Breast. Virchows Arch..

[51] Wang Z.-H., Pei X.-F., Zhu Z.-Q., Lin Z., Mao Y.-Y., Xu X.-L., Luo Y.-L., Zhang L., Peng P.-J. (2020). CD47 Overexpression Is Associated with Epstein–Barr Virus Infection and Poor Prognosis in Patients with Nasopharyngeal Carcinoma. Onco Targets Ther..

[52] Yu Y., Ke L., Xia W.-X., Xiang Y., Lv X., Bu J. (2019). Elevated Levels of TNF-α and Decreased Levels of CD68-Positive Macrophages in Primary Tumor Tissues Are Unfavorable for the Survival of Patients With Nasopharyngeal Carcinoma. Technol. Cancer Res. Treat..

[53] Chen Y.-L. (2020). Prognostic Significance of Tumor-Associated Macrophages in Patients with Nasopharyngeal Carcinoma. Medicine.

[54] Yu Y., Ke L., Lv X., Ling Y., Lu J., Liang H., Qiu W., Huang X., Liu G., Li W. (2018). The Prognostic Significance of Carcinoma-Associated Fibroblasts and Tumor-Associated Macrophages in Nasopharyngeal Carcinoma. Cancer Manag. Res..

[55] Deng R., Lu J., Liu X., Peng X.-H., Wang J., Li X.-P. (2020). PD-L1 Expression Is Highly Associated with Tumor-Associated Macrophage Infiltration in Nasopharyngeal Carcinoma. Cancer Manag. Res..

